# Evidence on Learning Style Preferences Among Clinical Students in Nigeria Using the Visual, Aural, Read/Write, and Kinesthetic Model: Cross-Sectional Study

**DOI:** 10.2196/84089

**Published:** 2026-07-10

**Authors:** Stella Ufuoma Rotifa, Ebi Bio Awotua-Efebo, Prayer Abu Owapiriba, Adedotun Daniel Adesina, Abisoye Sunday Oyeyemi, Aziba-anyam Gift Raimi, Christopher Ononiwu Elemuwa, Morufu Olalekan Raimi, Okechukwu Christian Oginifolunnia

**Affiliations:** 1 Department of Community Medicine, Federal Medical Centre Yenagoa, Yenagoa, Nigeria; 2 Department of Curriculum and Educational Technology, University of Port Harcourt, Port Harcourt, Nigeria; 3 Department of Sociology, University of Port Harcourt, Port Harcourt, Nigeria; 4 Department of Medical Services, Nigerian Law School, Yenagoa, Nigeria; 5 Department of Community Medicine, Faculty of Clinical Sciences, Niger Delta University, Wilberforce Island, Nigeria; 6 Department of Business Administration, Faculty of Management Sciences, Federal University Otuoke, Otuoke, Nigeria; 7 Department of Medical Microbiology, Parasitology and Immunology, Faculty of Medical Laboratory Sciences, Federal University Otuoke, Otuoke, Nigeria; 8 Niger Delta Institute for Emerging and Re-emerging Infectious Diseases, Federal University Otuoke, Flat 17b, Arizonal Street, Yenagoa, 560211, Nigeria, 23 47038053786; 9 Office of the State Coordinator, National Primary Healthcare Development Agency, Yenagoa, Nigeria

**Keywords:** clinical students, medical education, learning styles, visual, aural, read/write, and kinesthetic, VARK inventory, curriculum development, educational strategies, Nigeria, core Niger delta

## Abstract

**Background:**

Understanding how medical students learn is critical for improving teaching strategies in clinical education. Despite the widespread use of learning style frameworks, such as visual, aural, read/write, and kinesthetic (VARK), evidence from sub-Saharan Africa remains limited, and the use of learning style approaches is debated in the literature. In clinical and health sciences education, aligning teaching with learners’ preferences can enhance knowledge retention, procedural competence, and ultimately the quality of patient care.

**Objective:**

This study aimed to determine the predominant learning style preferences of clinical students at a Nigerian medical school and to examine how demographic and academic factors influence these preferences, with explicit attention to implications for clinical pedagogy.

**Methods:**

A cross-sectional survey was conducted among 200 clinical students (400-600 level) at Niger Delta University between October 2021 and December 2021, using the validated VARK inventory (version 7.8). Descriptive statistics summarized distributions, and the Pearson chi-square tests or Fisher exact tests assessed bivariate associations with sex, age group, and year of study. A multivariable modeling strategy was prespecified but not performed due to the categorical structure of the primary outcomes, sparse cells for some modality categories, and the sample size limitations for multinomial modeling.

**Results:**

Of 200 participants (mean age 25.1, SD 3.9 y; n=107, 53.5% male), 105 (52.5%) preferred unimodal learning, and 95 (47.5%) preferred multimodal learning. Kinesthetic (n=121, 60.5%) and auditory (n=110, 55%) were the most common dominant preferences, followed by read/write (n=68, 34%) and visual (n=36, 18%). Visual preference was significantly higher among male participants (χ^2^_1_=4.49; *P*=.03). Read/write preference varied by year of study (*χ*^2^_2_=8.29; *P*=.02). No significant associations were found with age. The pedagogical implications for clinical teaching were discussed, including bedside instruction, skills laboratory, simulation, small-group teaching, and audio-visual learning resources.

**Conclusions:**

Clinical students in this Nigerian setting predominantly favored kinesthetic and auditory learning, with nearly half reporting multimodal preferences. Medical educators should adopt blended instructional designs that include hands-on, discussion-based, and audio-visual elements to better prepare students for clinical practice. These insights can inform faculty development, curriculum design, and national medical education policies to foster adaptive, learner-centered training that improves clinical competency and readiness for professional service.

## Introduction

Learning styles refer to the distinctive methods individuals use to acquire and retain information, which ultimately shape the development of knowledge and skills [1-6]. Each student has a unique approach to learning, recalling information, and applying knowledge in different contexts [1,5,7-9]. These preferences significantly influence how much information a student absorbs and the time needed to do so [1,4,7]. In medical education, where the curriculum is both extensive and demanding, identifying and accommodating students’ learning preferences may improve knowledge retention and reduce resistance to instruction. Several models have been developed to conceptualize learning styles. The Dunn and Dunn model emphasizes 5 dimensions: environmental, emotional, sociological, physiological, and psychological, which collectively influence how students process information [3,10-12]. For example, environmental elements include lighting and sound, while physiological elements include sensory preferences and time of day. This framework highlights that individual and social differences, including gender and cultural background, shape students’ approaches to learning. Another widely used framework is the Fleming visual, auditory, read/write, and kinesthetic (VARK) model, developed in 1987 to help educators and students identify effective learning strategies [13]. VARK classifies learners as visual (preferring diagrams and illustrations), auditory (benefiting from spoken instruction and discussion), reading or writing (favoring text-based learning), or kinesthetic (learning best through hands-on experiences). By identifying these preferences, educators can adapt their teaching methods, and students can use strategies that align with their strengths. Other theories of learning, such as Kolb [14] experiential learning, the Grasha-Reichmann [15] learning style, Honey and Mumford [16] classification, the Felder-Silverman [17] model, and McCarthy [18] 4MAT system, also underscore the importance of aligning teaching with learning preferences [19-21]. However, in medical education, where students face heavy workloads and complex content, the VARK model has gained particular traction due to its simplicity and applicability in the classroom and clinical training [22].

In the context of medical and health sciences, learning preferences go beyond classroom performance; they influence the acquisition of clinical reasoning, procedural competence, communication, and teamwork, which are vital in patient care. Clinical students differ from preclinical learners because they must integrate theoretical knowledge with real-life patient interactions, skills laboratory sessions, and bedside teaching. Therefore, exploring learning styles among clinical students provides insights that can guide medical educators in designing teaching strategies that are both pedagogically sound and clinically relevant. While the VARK model is widely adopted, its predictive validity and pedagogical use remain debated. Systematic reviews and meta-analyses have cautioned against rigidly matching teaching to learning styles, emphasizing instead that effective medical education should incorporate multimodal, evidence-based instructional approaches that foster adaptability and critical thinking [23,24]. Nonetheless, understanding preferred modalities remains valuable for informing learner-centered curriculum design and promoting engagement, especially in resource-limited settings. There is a growing body of literature examining VARK learning preferences among medical students globally, with varying results. Studies from the United States, India, Saudi Arabia, and Malaysia report diverse modality distributions, with many students exhibiting multimodal preferences that combine VARK elements [25-34]. However, in sub-Saharan Africa, particularly Nigeria, evidence remains sparse and often limited to preclinical cohorts. Thus, understanding learning preferences is especially important in contexts such as Nigeria, where medical schools face challenges including large class sizes, limited teaching resources, and diverse student backgrounds. Prior studies have shown that accommodating students’ learning styles can improve academic outcomes, self-directed learning, and professional development [11,25-34]. However, few studies have focused specifically on clinical-year students who operate within high-stakes environments such as hospital wards and outpatient clinics, where experiential learning dominates. By focusing on this group, this study extends the scope of learning style research in Nigeria from theoretical instruction to applied clinical education. Despite this, limited research has examined the learning style preferences of clinical students in Nigerian medical schools, particularly in the Niger Delta region. Given the unique socioeconomic and infrastructural challenges in the region, such evidence is critical for optimizing medical training and aligning it with competency-based medical education reforms advocated by global health education frameworks [35,36]. This study, therefore, aimed to identify the dominant learning preferences of clinical students at Niger Delta University (NDU), Amassoma, using the VARK model. It also examined the influence of sex, age, and year of study on these preferences. The null hypothesis was that learning preferences do not differ by sex, age, or year of study, while the alternative hypothesis proposed that these factors do influence students’ preferred learning styles.

## Methods

### Study Area

The study was conducted at NDU, Amassoma, Southern-Ijaw Local Government Area, Bayelsa State, Nigeria. NDU is a state-owned university established in 2000 with multiple campuses, including the College of Health Sciences (CHS) in Amassoma and faculties spanning health sciences, basic medical sciences, nursing, pharmacy, sciences, agriculture, law, management sciences, social sciences, education, and arts and humanities. The CHS houses the clinical medicine programs, whose students (clinical years) form the target population for this study. The majority of students in the clinical phase at NDU originate from the southern region of Nigeria and are predominantly Christian; this sociodemographic context is noted to provide a relevant background for interpreting learning preference patterns in this cohort. The setting represents a typical resource-constrained Nigerian medical education environment, with limited simulation facilities, high student-to-tutor ratios, and diverse sociocultural backgrounds. These contextual features make it an appropriate setting for investigating learning style preferences among clinical medical students.

### Study Design

We used a cross-sectional, descriptive design to determine the distribution of learning preferences (VARK) and to explore associations between learning preferences and selected demographic factors (sex, age group, and year of study). This design was considered appropriate to capture a snapshot of learning preferences among clinical students during the specified study period. The cross-sectional design followed STROBE (Strengthening the Reporting of Observational Studies in Epidemiology) guidelines (Checklist 1) for observational studies to ensure methodological transparency and reproducibility.

### Study Population and Eligibility

The study population comprised all undergraduate clinical medical students in years 4, 5, and 6 in the CHS at NDU during the 2021 academic session. The nominal class populations were as follows: year 4 (n=94), year 5 (n=129), and year 6 (n=82; total N=200). Inclusion criteria included clinical students who had completed preclinical training and were currently in clinical rotations (years 4‐6). Students who had not completed preclinical requirements or who were unavailable during the data collection period were excluded. The selection of clinical students (rather than preclinical students) was intentional because clinical training emphasizes hands-on, experiential learning—an environment where learning style preferences directly influence skill acquisition, bedside learning, and clinical reasoning.

### Study Period

Data were collected between October 2021 and December 2021. The inclusion of this period ensures temporal context and allows for comparison with studies conducted in similar academic cycles.

### Sample Size Calculation

The minimum sample size was computed using the Cochran formula [37] for estimating a population proportion:


(1) $$n0\text{=}\text{\textbackslash{} }\frac{Z^{2}\text{\textbackslash{} }P\left(1\text{-}p\right)}{d^{2}}$$

where Z=1.96 (for 95% confidence), *P*=.88 (proportion of multimodal preference reported by Wehrwein et al [33]), 1-*P*=.125, and *d*=0.05 (margin of error). Substituting:


(2) $$n0\text{=}\text{\textbackslash{} }\frac{{\left(1.96\right)}^{2}\text{\textbackslash{} }\times \text{\textbackslash{} }0.875\text{\textbackslash{} }\times \text{\textbackslash{} }0.125}{{\left(0.05\right)}^{2}}$$

Allowing for 10% nonresponse, the adjusted sample size was calculated as 168 × 1.10=184.8≈185. To improve representativeness and generalizability, the sample size was rounded up, and the study recruited 200 students. This adjustment ensured adequate precision for detecting group differences (eg, across sex and year of study) at a power of approximately 0.80, which is consistent with recommendations for behavioral and educational research sample sizing [37].

### Sampling Technique and Sample Allocation

A proportionate, systematic random sampling approach was used. The sample of 200 was allocated to each class in proportion to the class population using the following equation:


(3) $$n_{i}\text{=}\left(\frac{N_{i}}{N_{total}}\right)\times n$$

where *N_i_* is the class population, *N_total_*=305, and n=200. The calculated allocations (before rounding) were: year 4 (n=61.64), year 5 (n=84.59), and year 6 (n=53.77). After rounding and a minor adjustment to ensure the final sample total equaled 200, the allocation used was: year 4 (n=62), year 5 (n=84), and year 6 (n=54). Within each class, the class register served as the sampling frame. A sampling interval k was computed for each class (*k*=*N_i_*/*n_i_*), a random start between 1 and *k* was selected, and every *k*th student on the register was invited. If a selected student declined or was unavailable, the next student on the register was approached as a replacement. Systematic random sampling was chosen to reduce selection bias while maintaining representativeness across all clinical years. This method is suitable for homogeneous academic cohorts and aligns with standard sampling recommendations for cross-sectional educational research [38].

### Study Instrument

Data were collected using a structured, self-administered questionnaire with two sections: (1) sociodemographic and educational data (age, sex, year of study, marital status, and residence) and the VARK inventory version 7.8 (the standard 16-item VARK questionnaire, where respondents can select one or more options per item to indicate preferred modalities [39-44]).

Scoring followed the official VARK protocol: each selected response scores 1 point in the corresponding modality; total scores across the 16 items determine each participant’s profile. Participants with a single highest score were classified as unimodal, while equal high scores in 2, 3, or 4 modalities were classified as bimodal, trimodal, and quadrimodal, respectively (multimodal). The VARK questionnaire (version 7.8) is a validated, internationally recognized tool that has demonstrated acceptable reliability across medical education settings. Its application in this study ensured comparability with global findings and reproducibility in similar low-resource academic contexts.

### Pretesting and Validity

The questionnaire was pretested among 20 year-3 (preclinical) students at NDU to assess clarity, comprehension, and administration logistics. Content validity was assessed through consultation with subject-matter experts in medical education and instrument design; feedback informed language edits and minor item clarifications before the main study. Internal consistency (reliability) of the VARK items in the pilot was evaluated using Cronbach α. The scale demonstrated acceptable reliability, with a Cronbach *α* of 0.78. Item-total correlations ranged from 0.42 to 0.68, indicating that each item contributed meaningfully to the overall scale reliability [45-51]. The pretest confirmed cultural appropriateness and face validity for the Nigerian clinical student context, ensuring that terminologies and examples were easily comprehensible. This process aligns with best practices for instrument adaptation in cross-cultural educational research [52].

### Data Collection and Quality Control

Three resident doctors from the Department of Community Medicine, Federal Medical Center, Yenagoa, were recruited and trained as research assistants. The training spanned 3 sessions (total of 6 h) and covered study objectives, ethical conduct, informed consent procedures, questionnaire administration, and completeness checks. Data collectors approached students during scheduled breaks, explained the study, obtained written informed consent, and distributed the self-administered questionnaires. Completed questionnaires were reviewed immediately for completeness; respondents with missing items were politely asked to complete them where possible. Data were entered into Microsoft Excel and cleaned before analysis. Data cleaning included range and consistency checks. To ensure accuracy, a double-entry procedure was used: 2 independent research assistants entered all questionnaire data separately. The 2 datasets were then cross-checked, and any discrepancies were flagged. Discrepancies were resolved by referring back to the original questionnaires, with the principal investigator making the final determination. This process minimized transcription errors and ensured a high level of data integrity. A double-entry verification process and regular data audits were instituted to enhance accuracy and reproducibility, consistent with data integrity standards. No personally identifiable information was stored with the responses.

### Statistical Analysis

Data were analyzed using IBM SPSS version 24. Continuous variables were summarized as mean (SD) or median (IQR), as appropriate, while categorical variables were presented as counts and percentages. Associations between categorical predictors (sex, age group, and year) and VARK outcomes were tested using the Pearson chi-square test or Fisher exact test, where assumptions were violated. Exact *P* values are reported. A multivariable modeling approach (binary logistic for multimodal vs unimodal; multinomial logistic for modal categories) was prespecified in the protocol. During analysis, we assessed the feasibility of adjusted modeling: given the large number of modal categories (many with small cell counts) and the overall sample size (N=200), which produced sparse cells for several modality categories, robust multinomial models would risk unstable estimates and overfitting. For the specific contrast of multimodal versus unimodal, candidate predictors were limited; however, when we attempted exploratory binary logistic models, the predictors showed unstable parameter estimates and wide CIs due to sparse cells. After careful consideration (and to avoid presenting potentially misleading adjusted estimates), we decided to report the complete bivariate results and explicitly note in the Methods and Results sections that adjusted multivariable models were not performed for the reasons stated. Exploratory models yielded inflated SEs and wide CIs. To maintain statistical robustness and transparency, only the bivariate analyses are reported. The rationale for omitting multivariable models is stated explicitly in both the Methods and Results sections. This decision aligns with best practice guidance for small-sample categorical analyses [53] and avoids overfitting while ensuring valid inference.

### Ethical Considerations

Ethical approval was obtained from the Research Ethics Committee, NDU, Amassoma (NDUREC/2021/210). The study was conducted in accordance with the Declaration of Helsinki. Participants provided written informed consent before participation. Data were anonymized at the point of entry by removing personal identifiers and were stored on password-protected computers accessible only to the research team. Participants were assured of confidentiality, anonymity, and voluntary participation, with the option to withdraw at any point without academic consequence. Ethical procedures also conformed to the Nigerian National Health Research Ethics Code [54] and JMIR’s ethical reporting policy. Compensation was not provided for participation.

## Results

### Participant Characteristics

Table 1 presents an overview of the demographic characteristics of the study participants. Of the 200 participants, 107 (53.5%) were male and 93 (46.5%) were female. The mean age was 25.0 (SD 3.9) years. Most students were aged 21 to 25 years (n=109, 54.5%), followed by those aged 25 to 29 years (n=78, 39%), while 13 (6.5%) were 30 years or older. The majority were single (n=151, 75.5%), with 49 (24.5%) married or cohabiting. Regarding residence, 73 (36.5%) lived on campus and 127 (63.5%) lived off campus. By year of study, 62 (31%) were in the 400 level, 85 (42.5%) in the 500 level, and 53 (26.5%) in the 600 level. Most students reported attending lectures 5 times weekly (n=186, 93%), while 14 (7%) attended 3 to 4 times weekly.

**Table 1.** Sociodemographic and academic characteristics of clinical students (N=200).

Characteristic	Values, n (%)
Sex	
Male	107 (53.5)
Female	93 (46.5)
Age group (y)	
21‐25	109 (54.5)
25‐29	78 (39)
≥30	13 (6.5)
Marital status	
Single	151 (75.5)
Married or cohabiting	49 (24.5)
Resident of participant	
On campus	73 (36.5)
Off campus	127 (63.5)
Year of study	
400 level	62 (31)
500 level	85 (42.5)
600 level	53 (26.5)
Frequency of lecture attendance	
5 times	186 (93)
3-4 times	14 (7)

### Learning Style Dominance and Learning Modality Among Clinical Students in NDU

**Figure 1.**
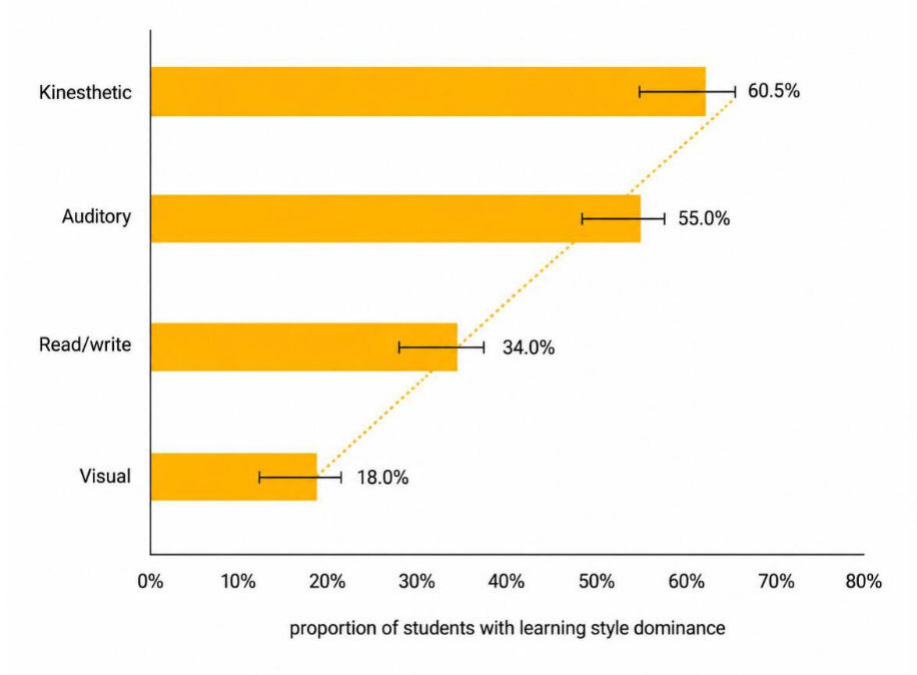
Learning style dominance among clinical students at Niger Delta University (NDU), Amassoma.

Table 2, Figure 1, and Multimedia Appendix 1 provide a detailed breakdown of the preferred learning styles among clinical students at NDU, Amassoma, highlighting both unimodal and multimodal preferences. Among unimodal learners, 48 (24%) preferred kinesthetic, 33 (16.5%) auditory, 21 (10.5%) read/write, and 3 (1.5%) visual. For bimodal learners, the most frequent combination was auditory-kinesthetic (n=28, 14%), followed by read/write-kinesthetic (n=13, 6.5%) and auditory-read/write (n=12, 6%). A small group (n=6, 3%) reported quadrimodal preferences. Overall, kinesthetic (n=121, 60.5%) and auditory (n=110, 55%) were the most commonly reported dominant preferences. In modality classification, 105 (52.5%) were unimodal and 95 (47.5%) multimodal (Table 2, Figure 1, and Multimedia Appendix 1).

**Table 2.** Learning style preference and modality among clinical students at Niger Delta University (NDU; N=200).

Learning style preference	Modality	Values, n (%)
Kinesthetic	Unimodal	48 (24)
Auditory	Unimodal	33 (16.5)
Read/write	Unimodal	21 (10.5)
Visual	Unimodal	3 (1.5)
AK^a^	Bimodal	28 (14)
RK^b^	Bimodal	13 (6.5)
AR^c^	Bimodal	12 (6)
VA^d^	Bimodal	7 (3.5)
VK^e^	Bimodal	3 (1.5)
VR^f^	Bimodal	1 (0.5)
ARK^g^	Trimodal	11 (5.5)
VAK^h^	Trimodal	10 (5)
VAR^i^	Trimodal	2 (1)
VRK^j^	Trimodal	2 (1)
VARK^k^	Quadrimodal	6 (3)

a AK: auditory and kinesthetics.

b RK: read/write and kinesthetics.

c AR: auditory and read/write.

d VA: visual and auditory.

e VK: visual and kinesthetic.

f VR: visual and read/write.

g ARK: auditory, read/write, and kinesthetic.

h VAK: visual, auditory, and kinesthetic.

i VAR: visual, auditory, and read/write.

j VRK: visual, read/write, and kinesthetic.

k VARK: visual, auditory, read/write, and kinesthetic.

### Association Between Gender and Dominant Learning Preferences or Modalities of the Students

**Figure 2.**
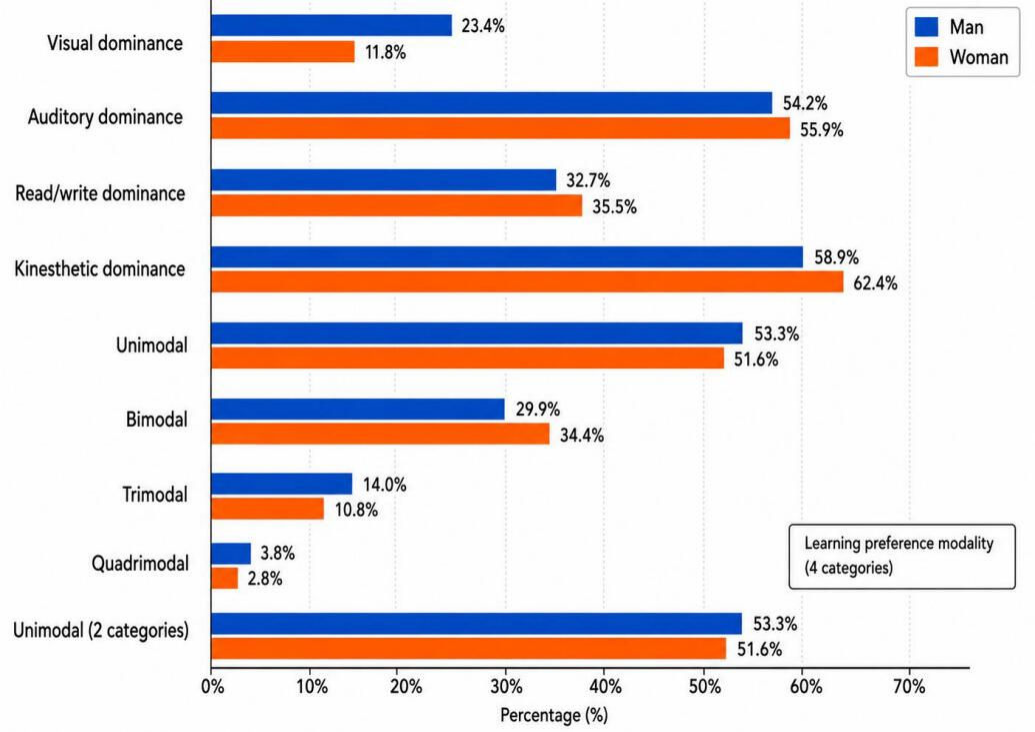
A grouped bar chart showing the distribution of learning preferences by gender.

Table 3 and Figure 2 present an in-depth analysis of learning style preferences among clinical students at NDU, Amassoma, stratified by gender. The data reveal notable differences and similarities in learning preferences between male and female students. Visual learning preference was significantly higher among males (25/107, 23.4%) compared with females (11/93, 11.8%; *χ*^2^_1_=4.49; *P*=.03). No significant gender differences were found for auditory (58/107, 54.2% males vs 52/93, 55.9% females), read/write (35/107, 32.7% males vs 33/93, 35.5% females), or kinesthetic (63/107, 58.9% males vs 58/93, 62.4% females) preferences (*P*>.05). For learning modality, unimodal preference was observed in 57 out of 107 (53.3%) males and 48 out of 93 (51.6%) females, while multimodal preference was observed in 50 out of 107 (46.7%) males and 45 out of 93 (48.4%) females. These differences were not statistically significant (*χ*^2^_3_=0.79; *P*=.85).

**Table 3.** Association between gender and dominant learning preference of the students (N=200).

Learning preference	Total, N (%)	Men, n (%)	Women, n (%)	*χ*^2^ (*df*)	*P* value
Visual dominance				4.49 (1)	.03^a^
Yes	36 (18.0)	25 (23.4)	11 (11.8)		
No	164 (82.0)	82 (76.6)	82 (88.2)		
Auditory dominance				0.06 (1)	.81
Yes	110 (55.0)	58 (54.2)	52 (55.9)		
No	90 (45.0)	49 (45.8)	41 (44.1)		
Read/write dominance				0.17 (1)	.68
Yes	68 (34.0)	35 (32.7)	33 (35.5)		
No	132 (66.0)	72 (67.3)	60 (64.5)		
Kinesthetic dominance				0.25 (1)	.62
Yes	121 (60.5)	63 (58.9)	58 (62.4)		
No	79 (39.5)	44 (41.1)	35 (37.6)		
Learning preference modality (4 categories)				0.79 (3)	.85
Unimodal	105 (52.5)	57 (53.3)	48 (51.6)		
Bimodal	64 (32.0)	32 (29.9)	32 (34.4)		
Trimodal	25 (12.5)	15 (14.0)	10 (10.8)		
Quadrimodal	6 (3.0)	3 (2.8)	3 (3.2)		
Learning preference modality (2 categories)				0.06 (1)	.82
Unimodal	105 (52.5)	57 (53.3)	48 (51.6)		
Multimodal	95 (47.5)	50 (46.7)	45 (48.4)		

a Statistically significant at *P* values <.05.

### Association Between the Age of Participants and the Dominant Learning Preferences or Modalities of the Students

**Figure 3.**
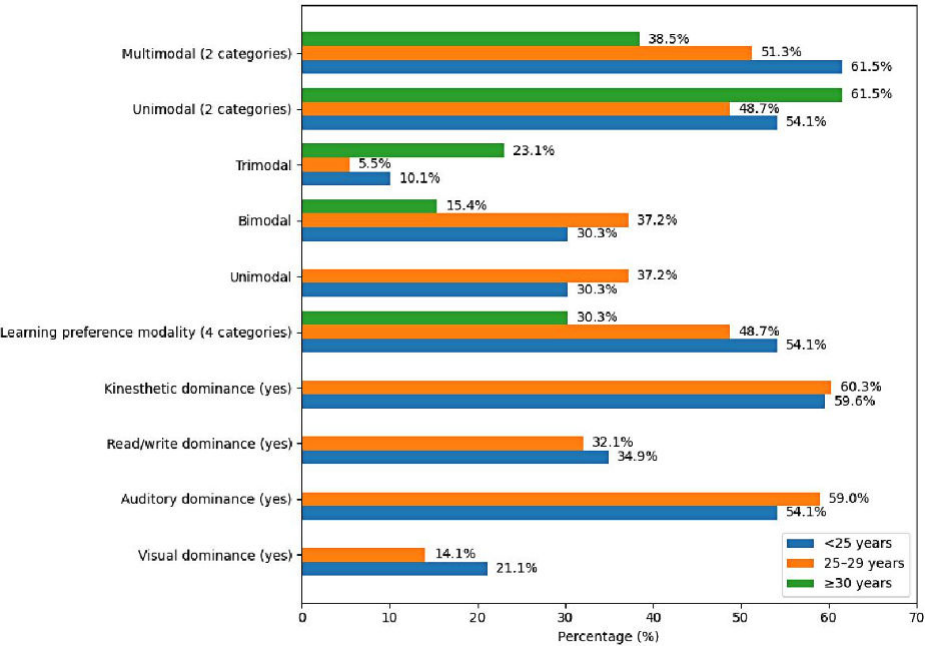
A grouped bar chart showing the distribution of each learning preference across the 3 age groups (<25, 25‐29, and ≥30 years).

Table 4 and Figure 3 provide a detailed analysis of learning style preferences among clinical students at NDU, Amassoma, categorized by age groups. The data highlight various trends in learning preferences across different age cohorts. Visual dominance was most common among students younger than 25 years (23/109, 21.1%). Auditory preference peaked in the 25 to 29 years group (46/78, 59.0%), while read/write (5/13, 38.5%) and kinesthetic (9/13, 69.2%) preferences were most frequent among those aged 30 years or older. None of these differences were statistically significant (*P*>.05). For learning modality, unimodal preference was highest in the 30 years or older group (8/13, 61.5%), whereas multimodal preference was most common in the 25 to 29 years group (40/78, 51.3%). Trimodal learning occurred in 3 of 13 (23.1%) of those aged 30 years or older, while quadrimodal learning was reported in 6 of 109 (5.5%) of students younger than 25 years. No significant associations were found between age and learning modality (*χ*^2^_6_=9.19, *P*=.16 for unimodal; *χ*^2^_2_=0.98, *P*=.61 for multimodal).

**Table 4.** Association between learning preference dominance and the age of the students (N=200).

Learning preference	Total, N (%)	Aged <25 years, n (%)	Aged 25‐29 years, n (%)	Aged ≥30 years, n (%)	*χ*^2^ (*df*)	*P* value
Visual dominance					1.57 (2)	.46
Yes	36 (18.0)	23 (21.1)	11 (14.1)	2 (15.4)		
No	164 (82.0)	86 (78.9)	67 (85.9)	11 (84.6)		
Auditory dominance					1.98 (2)	.37
Yes	110 (55.0)	59 (54.1)	46 (59.0)	5 (38.5)		
No	90 (45.0)	50 (45.9)	32 (41.0)	8 (61.5)		
Read/write dominance					0.28 (2)	.87
Yes	68 (34.0)	38 (34.9)	25 (32.1)	5 (38.5)		
No	132 (66.0)	71 (65.1)	53 (67.9)	8 (61.5)		
Kinesthetic dominance					0.45 (2)	.80
Yes	121 (60.5)	65 (59.6)	47 (60.3)	9 (69.2)		
No	79 (39.5)	44 (40.4)	31 (39.7)	4 (30.8)		
Learning preference modality (4 categories)					9.19 (6)	.16
Unimodal	105 (52.5)	59 (54.1)	38 (48.7)	8 (61.5)		
Bimodal	64 (32.0)	33 (30.3)	29 (37.2)	2 (15.4)		
Trimodal	25 (12.5)	11 (10.1)	11 (14.1)	3 (23.1)		
Quadrimodal	6 (3.0)	6 (5.5)	0 (0.0)	0 (0.0)		
Learning preference modality (2 categories)					0.98 (2)	.61
Unimodal	105 (52.5)	59 (54.1)	38 (48.7)	8 (61.5)		
Multimodal	95 (47.5)	50 (45.9)	40 (51.3)	5 (38.5)		

### Association Between Year of Study and Dominant Learning Preferences or Modalities of the Students

**Figure 4.**
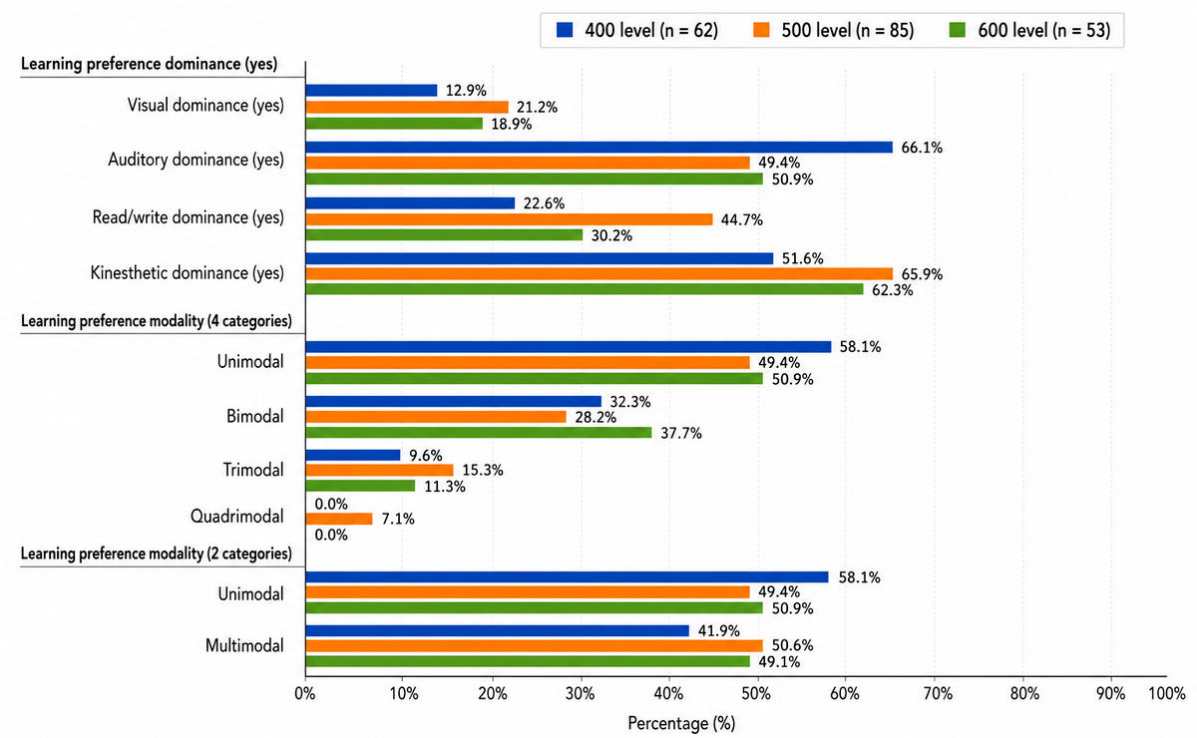
A grouped bar chart showing the learning preferences of clinical students by year of study. It compares 400-, 500-, and 600-level students across all learning preference categories.

Table 5 and Figure 4 provide an insightful breakdown of learning style preferences among clinical students at NDU, Amassoma, categorized by their respective academic years. The data highlight various trends in learning preferences across different stages of their educational journey. Visual dominance was reported by 8 out of 62 (12.9%) 400-level students, 18 out of 85 (21.2%) 500-level students, and 10 out of 53 (18.9%) 600-level students (*χ*^2^_2_=1.69; *P*=.43). Auditory preference was highest in 400-level students (41/62, 66.1%) compared with 500-level (42/85, 49.4%) and 600-level (27/53, 50.9%) students (*P*>.05). Kinesthetic preference ranged from 32 of 62 (51.6%) in 400-level students to 56 of 85 (65.9%) in 500-level students and 33 of 53 (62.3%) in 600-level students, also not significant (*P*>.05). Read/write preference varied significantly across years (*χ*^2^_2_=8.29; *P*=.02): 14 out of 62 (22.6%) in 400-level, 38 out of 85 (44.7%) in 500-level, and 16 out of 53 (30.2%) in 600-level. For modality classification, unimodal learning was most common in 400-level students (36/62, 58.1%), bimodal in 600-level students (20/53, 37.7%), and trimodal in 500-level students (13/85, 15.3%). Quadrimodal preference was reported only among 500-level students (6/85, 7.1%). No significant associations were found between the year of study and learning modality (*P*>.05).

**Table 5.** Association between the year of study and the learning preference dominance of the students (N=200).

Learning preference	Total, N (%)	400 level, n (%)	500 level, n (%)	600 level, n (%)	*χ*^2^ (*df*)	*P* value
Visual dominance					1.69 (2)	.43
Yes	36 (18.0)	8 (12.9)	18 (21.2)	10 (18.9)		
No	164 (82.0)	54 (87.1)	67 (78.8)	43 (81.1)		
Auditory dominance					4.53 (2)	.10
Yes	110 (55.0)	41 (66.1)	42 (49.4)	27 (50.9)		
No	90 (45.0)	21 (33.9)	43 (50.6)	26 (49.1)		
Read/write dominance					8.29 (2)	.02^a^
Yes	68 (34.0)	14 (22.6)	38 (44.7)	16 (30.2)		
No	132 (66.0)	48 (77.4)	47 (55.3)	37 (69.8)		
Kinesthetic dominance					3.15 (2)	.21
Yes	121 (60.5)	32 (51.6)	56 (65.9)	33 (62.3)		
No	79 (39.5)	30 (48.4)	29 (34.1)	20 (37.7)		
Learning preference modality (4 categories)					10.57 (6)	.10
Unimodal	105 (52.5)	36 (58.1)	42 (49.4)	27 (50.9)		
Bimodal	64 (32.0)	20 (32.3)	24 (28.2)	20 (37.7)		
Trimodal	25 (12.5)	6 (9.6)	13 (15.3)	6 (11.3)		
Quadrimodal	6 (3.0)	0 (0.0)	6 (7.1)	0 (0.0)		
Learning preference modality (2 categories)					1.15 (2)	.56
Unimodal	105 (52.5)	36 (58.1)	42 (49.4)	27 (50.9)		
Multimodal	95 (47.5)	26 (41.9)	43 (50.6)	26 (49.1)		

a Statistically significant at *P*<.05.

## Discussion

### Principal Findings

In this study, kinesthetic emerged as the most common learning preference among both male and female clinical students, while visual preference was the only modality significantly associated with gender, being higher among males. Nearly half of all students demonstrated multimodal learning tendencies, underscoring the diverse cognitive and sensory strategies adopted in clinical education. These findings reinforce the importance of adopting multimodal instructional approaches in medical training to accommodate the broad spectrum of learning preferences.

### Gender-Based Variations in Learning Styles

The predominance of kinesthetic learning across both sexes contrasts with studies in the United States and India, which reported broader gender differences across multiple modalities [39,55]. For example, Wehrwein et al [39] at Michigan State University found that most females preferred unimodal learning styles (especially kinesthetic), while males exhibited a higher proportion of multimodal preferences. Similarly, research in Kota, India [55] revealed that both male and female medical students preferred multimodal learning, but the degree of preference varied significantly between genders. These discrepancies likely reflect differences in teaching cultures and curricular design. At NDU, where traditional didactic lectures dominate, exposure to multimodal pedagogies such as simulation or problem-based learning is limited. Consequently, learning style self-reports may mirror exposure rather than inherent cognitive tendencies. This study’s findings suggest that educational context, rather than sex alone, may play a larger role in shaping learning preferences. By introducing interactive, experiential teaching strategies, such as simulation and case-based learning, educators can create more inclusive environments that bridge these gender-linked variations.

### Influence of Age on Learning Preferences

The age of participants did not significantly influence learning style preferences, though minor variations were observed across age groups. This aligns with a systematic review of VARK-based studies in Nepal, which similarly concluded that age bore no clear association with learning preferences [13]. Conversely, Mohammadi et al [56] at Iran Medical School found both age and sex to be significant predictors of learning modalities. The discrepancy between these studies may arise from cultural, curricular, or experiential differences across institutions. In our cohort, the relatively narrow age range (predominantly 21‐26 y) among clinical students likely reduced variability in learning exposure and maturity, explaining the nonsignificant age effects. Overall, age appears to be a contextual moderator rather than a determinant of learning style; its influence may be more pronounced in heterogeneous samples that include postgraduate or continuing education learners.

### Relationship Between Level of Study and Learning Modalities

A notable finding was the read/write dominance among 500-level students (44.7%), compared to auditory dominance in 400-level students and kinesthetic preference in 600-level students. This trend may indicate a progression from theoretical to practical learning preferences as students transition through clinical training. This contrasts with findings from a Saudi Arabian dental college, where 24.2% of clinical students showed visual dominance [57]. The divergence could stem from differences in instructional technology and visual learning aids available in Saudi Arabia compared with Nigeria’s resource-constrained context. Our results suggest that as students gain hands-on experience, their learning preferences shift toward kinesthetic and multimodal approaches. This aligns with experiential learning theory, which posits that practical exposure enhances the ability to integrate sensory modalities. Parallel evidence from Daud et al [58] in Pakistan showed an increasing preference for multimodal styles as students advanced academically. Similarly, Marzo et al [59] reported that 54.9% of clinical students in Malaysia preferred multiple learning modes, compared with 53.6% unimodal preferences among preclinical students. These findings collectively affirm that learning preferences evolve with curricular exposure and underscore the need for year-specific pedagogical adaptation.

### Global and Regional Comparisons

Studies in Saudi Arabia, India, Turkey, and Sri Lanka consistently demonstrate that kinesthetic and multimodal preferences are predominant among medical students [55-63]. For instance, Baykan and Nacar [61] found that 63.9% of Turkish students were multimodal learners, while Samarakoon et al [4] in Colombo reported multimodal preferences among 69.9% of preclinical and 67.5% of clinical students. In contrast, US-based studies (eg, Wehrwein et al [39]) show greater heterogeneity, with female students favoring unimodal kinesthetic approaches and male students preferring multimodal approaches. This pattern may reflect greater curricular flexibility, simulation exposure, and student-centered learning environments in developed contexts, factors still emerging in many African and Asian medical schools. The limited availability of high-quality comparative data from sub-Saharan Africa restricts the generalizability of findings. Few published African studies have systematically examined VARK preferences among clinical students, making this research one of the first locale-specific contributions from the Niger Delta region.

### Pedagogical Implications for Clinical Education

Beyond identifying learning styles, the practical relevance lies in how educators respond to them. Medical education, particularly in resource-limited settings, must balance traditional didactic instruction with experiential and technology-enhanced modalities. Our findings suggest that a blended pedagogical framework integrating lectures, simulation, small-group discussions, and visual aids would most effectively address the diversity of learning preferences as follows: (1) kinesthetic learners benefit from skills-lab demonstrations, bedside teaching, and simulation-based education; (2) auditory learners thrive in interactive case discussions and team-based learning; (3) visual learners need charts, clinical imaging, and structured Microsoft PowerPoint visuals; and (4) read/write learners perform best with concise study guides and evidence summaries.

By embedding these multimodal elements into the curriculum, medical educators can improve knowledge retention, student engagement, and self-directed learning, which are key competencies in clinical practice. While our findings add to regional evidence, several methodological considerations must be noted. The VARK inventory captures self-reported preferences, which may not always reflect actual learning behavior in clinical settings. Additionally, cultural interpretations of “learning” may influence how students rate items, introducing subtle bias. The study was conducted in a single institution with a modest sample size, limiting generalizability. Furthermore, multivariable modeling was not performed due to sparse data in certain categories; thus, inferences are limited to bivariate associations. Despite these limitations, the use of a validated instrument (VARK version 7.8), rigorous sampling, and quality control measures strengthens internal validity. The study also provides much-needed contextual data from a sub-Saharan African clinical education environment, filling a documented gap in the literature.

### Integrating Findings With Broader Literature

The discussion of learning styles must be situated within ongoing debates about their pedagogical utility. Systematic reviews have questioned the efficacy of matching instruction to preferred modalities, suggesting that learning outcomes depend more on engagement strategies, feedback, and active learning [23,24,64-70]. Thus, while recognizing students’ learning preferences can improve motivation and participation, educators should avoid rigid “style-matching” and instead promote adaptive, multimodal teaching that enhances clinical competence. This study’s results contribute to a growing movement advocating for “learning flexibility” rather than “learning fixation,” especially in clinical contexts where multimodal integration mirrors real-world medical practice. Overall, this study reaffirms the diversity and dynamism of learning preferences among clinical medical students. Kinesthetic and auditory modes dominate, but multimodal tendencies highlight the need for flexible, student-centered teaching. Future research should adopt multicenter, longitudinal, and mixed methods designs to explore how learning preferences evolve with clinical experience and to validate these patterns across different educational systems.

### Limitations

This study has several limitations. First, learning preferences were assessed through self-reported questionnaires (VARK), which may be subject to recall and social desirability bias. Reported preferences may not fully reflect students’ actual learning behaviors in practice. Although the VARK inventory (version 7.8) is a validated instrument widely used in medical education, it measures perceived rather than demonstrated learning tendencies. Future studies could complement VARK scores with observational or performance-based assessments to better triangulate actual learning behavior. Second, the study was conducted at a single institution, NDU, Amassoma, which may limit generalizability. Findings may not represent medical students in other Nigerian universities or international contexts with different curricular structures, teaching methods, or resource levels. However, given that NDU’s medical curriculum follows the standard Nigerian Medical and Dental Council framework, the findings remain contextually relevant to many similar resource-constrained medical schools across sub-Saharan Africa. Third, the sample included a relatively small number of students aged 30 years or older (n=13), reducing the statistical power to detect age-related differences in learning preferences. This age imbalance reflects the typical demographic profile of Nigerian medical students, who often enter clinical training shortly after secondary or undergraduate studies. Nonetheless, the limited age diversity restricts the exploration of learning style variability across maturity levels. Fourth, the cross-sectional design precludes conclusions about how learning preferences may evolve as students progress through medical training. A longitudinal approach would allow for tracking changes in modality preferences over time, especially as students transition from preclinical to clinical phases and gain more experiential exposure. Additionally, while the study used a proportionate, systematic sampling technique and achieved a robust sample size (N=200), potential selection bias cannot be entirely ruled out if students with particular learning preferences were more inclined to participate. Furthermore, the study did not assess academic performance outcomes, which could have provided insight into how learning preferences relate to achievement. Despite these limitations, the study provides novel and context-specific insights into the learning preferences of clinical medical students in a Nigerian setting. By identifying modality trends and demographic influences within a resource-limited educational environment, this study lays foundational evidence for future research on adaptive teaching strategies in medical education. Future research could employ longitudinal and multicenter designs, incorporating multiple institutions across different regions, and integrate objective measures of learning behaviors (eg, performance metrics and simulation outcomes) alongside self-reports. Such designs would improve both internal and external validity, enhance the robustness of findings, and support the development of evidence-based, learner-centered curricula tailored to diverse educational contexts.

### Recommendations

Based on the study’s findings and in line with current best practices in medical and health sciences education, the following recommendations are proposed to enhance teaching and learning among clinical students at NDU and comparable institutions:

- Tailored instructional strategies: medical educators should integrate a variety of teaching methods that address diverse learning styles, particularly the kinesthetic and auditory preferences identified among clinical students. This includes *hands-on activities*, *simulation-based learning*, *bedside demonstrations*, *case-based tutorials*, and *peer-assisted teaching**,*** which promote active engagement and deeper understanding. Such approaches are consistent with competency-based medical education principles that emphasize experiential learning, psychomotor skill development, and reflective practice.
- Diversification of teaching approaches: beyond traditional didactic lectures, medical schools should diversify pedagogical strategies by incorporating small group discussions, flipped classroom models, blended e-learning modules, and multimedia-based instruction (eg, instructional videos, podcasts, and interactive virtual simulations). These techniques not only accommodate multimodal learners but also enhance critical thinking, problem-solving, and communication skills essential for clinical competence. They are particularly beneficial in resource-limited settings where flexibility and innovation can offset infrastructural constraints.
- Inclusive and adaptive curriculum development: curriculum designers should ensure that instructional content and delivery methods reflect the diversity of student learning preferences. Flexibility and inclusivity can be achieved by embedding learning style awareness modules within faculty training and by structuring assessments that evaluate knowledge application across modalities. Curriculum review committees should incorporate student feedback mechanisms and educational research evidence to continuously align teaching strategies with evolving learner needs. Embedding inclusivity aligns with Sustainable Development Goal 4 (quality education), promoting equity and access in medical training.
- Longitudinal and outcome-based evaluation: future studies should assess the long-term effects of tailored instructional strategies on students’ learning outcomes, professional development, and clinical competencies. This should include longitudinal cohort studies tracking changes in learning preferences as students transition from preclinical to clinical training phases, linking these trends with academic performance and patient-care readiness. Incorporating mixed methods approaches, combining quantitative VARK analysis with qualitative student reflections, would yield richer insights.
- Cross-institutional and comparative research: collaborative, multicenter studies across Nigerian and international medical schools are encouraged to explore contextual and cultural influences on learning preferences. Comparative regional and global research could identify universal and culture-specific patterns in learning preferences, helping to standardize adaptive educational policies. Partnerships among universities can also facilitate shared best practices in instructional design, teacher training, and digital education infrastructure.
- Continuous professional development for educators: medical educators should be regularly exposed to faculty development workshops, seminars, and mentorship programs on evidence-based teaching and learning strategies. Continuous professional development programs should specifically address the application of adult learning theories, the use of educational technology, and the integration of student-centered pedagogies into medical curricula. Creating institutional teaching excellence centers could sustain professional growth and innovation.
- Policy and accreditation alignment: National accreditation bodies and policymakers should encourage the inclusion of multimodal, learner-centered pedagogy within medical education standards. Embedding learning style considerations into accreditation checklists and national benchmarks will promote educational consistency, equity, and innovation across medical schools in Nigeria and sub-Saharan Africa.

Implementing these recommendations can contribute to the continuous improvement of medical education programs, ensuring they remain responsive to the evolving needs of students and the health care sector. Ultimately, aligning instructional practices with empirically derived learning profiles can enhance knowledge retention, skill acquisition, and holistic competence among future health care professionals, thereby improving patient outcomes and strengthening the overall health system capacity.

### Significance Statement

**Figure 5.**
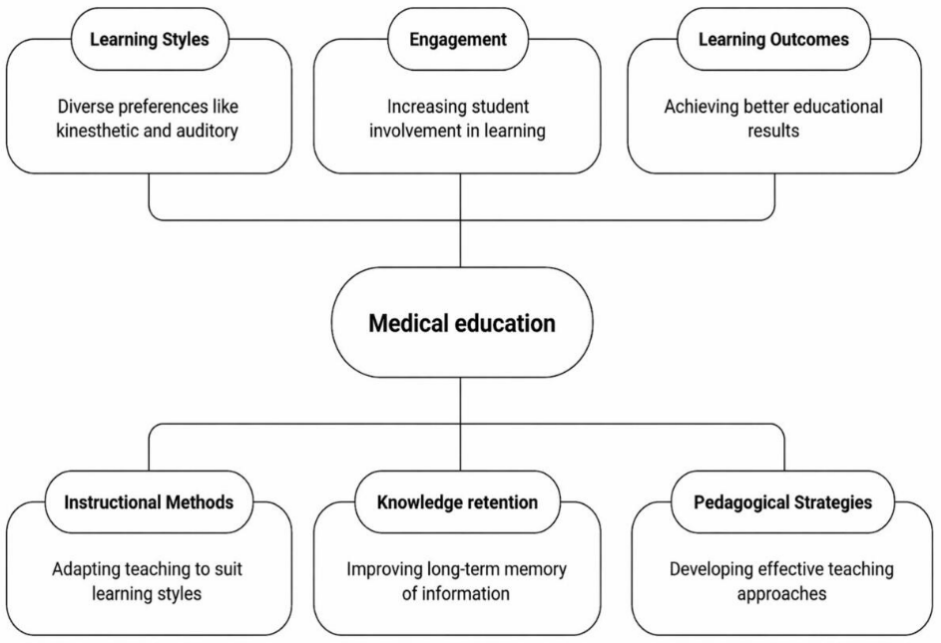
Enhancing medical education through learning styles.

This study holds significant implications for medical education by highlighting the diverse learning preferences among clinical students at NDU, Amassoma. By identifying predominant learning styles such as kinesthetic and auditory preferences, the study underscores the importance of adapting instructional methods to accommodate these preferences. This personalized approach can enhance engagement, improve knowledge retention, and ultimately optimize learning outcomes in medical education. Moreover, the study contributes to the body of knowledge on learning styles in medical education, particularly in the context of resource-constrained environments like Nigeria. By recognizing the prevalence of specific learning styles among students, educators and curriculum developers gain insights that can inform pedagogical strategies aimed at maximizing educational effectiveness. This understanding is crucial for fostering a supportive learning environment that meets the diverse needs of learners. Furthermore, the findings serve as a foundation for future research endeavors in medical education. They provide a basis for exploring how different instructional strategies tailored to specific learning styles can impact student performance and clinical competency development over the course of their medical training. Such research could lead to innovations in curriculum design and teaching methodologies that better prepare medical students for the challenges of modern health care practice. This is graphically represented in Figure 5.

### Conclusions

This study highlights the diverse learning style preferences among clinical medical students at NDU, Amassoma. While kinesthetic and auditory modalities emerged as the most common, visual and read/write preferences were also evident, underscoring the heterogeneity within the cohort. Gender differences were limited to visual learning, which was significantly more common among males, whereas other modalities showed no meaningful variation. Age did not significantly influence learning preferences, and the only notable year-level effect was the higher prevalence of read/write preference among 500-level students. The findings contribute to the broader understanding of medical education in resource-constrained contexts, where teaching practices are often dominated by lecture-based instruction. By empirically documenting the distribution of learning preferences, this study offers locally relevant data that can inform evidence-based pedagogical reforms in Nigeria and similar sub-Saharan settings. These findings suggest that medical educators should avoid one-size-fits-all approaches and instead provide varied, flexible instructional strategies that engage multiple sensory modalities. From a practical standpoint, the curriculum should integrate more hands-on activities, such as skills laboratory simulations, bedside teaching, and patient demonstrations, to accommodate kinesthetic learners. Additionally, it should incorporate tutorials, group discussions, and audio-visual resources to support auditory and visual learners. Curriculum planners and policy developers should also consider embedding structured faculty development programs that train educators to recognize and respond to learning diversity. These programs can help align instructional delivery with evidence-based approaches and national medical education standards. At the policy level, institutions and accreditation bodies may integrate multimodal and learner-centered pedagogies into competency-based medical curricula. Doing so will not only improve learning engagement but may also enhance long-term clinical reasoning, teamwork, and communication skills essential to patient care. Moving beyond traditional didactic lectures toward a more interactive, blended learning model will better cater to the diverse range of student preferences. By acknowledging and accommodating these differences, educators and curriculum developers can foster a more inclusive learning environment, enhance student engagement, and ultimately strengthen the effectiveness of medical training. Future research should expand this evidence base by linking learning style profiles to academic performance and clinical competence outcomes. Such evidence will support data-driven reforms in medical pedagogy and contribute to the global discourse on how to best prepare health professionals for the complexities of 21st-century health care delivery.

## Supplementary material

10.2196/84089 Multimedia Appendix 1 Learning modalities among clinical students at Niger Delta University (NDU), Amassoma.

10.2196/84089 Checklist 1 STROBE checklist.

## Abbreviations

CHS: College of Health Sciences
NDU: Niger Delta University
STROBE: Strengthening the Reporting of Observational Studies in Epidemiology
VARK: visual, auditory, read/write, and kinesthetic
