# Supplementary figures and images for: Evidence on Learning Style Preferences Among Clinical Students in Nigeria Using the Visual, Aural, Read/Write, and Kinesthetic Model: Cross-Sectional Study
Source: JMIR Form Res. 2026 Jul 10;10:e84089. doi: 10.2196/84089 (PMC13359485; doi:10.2196/84089)

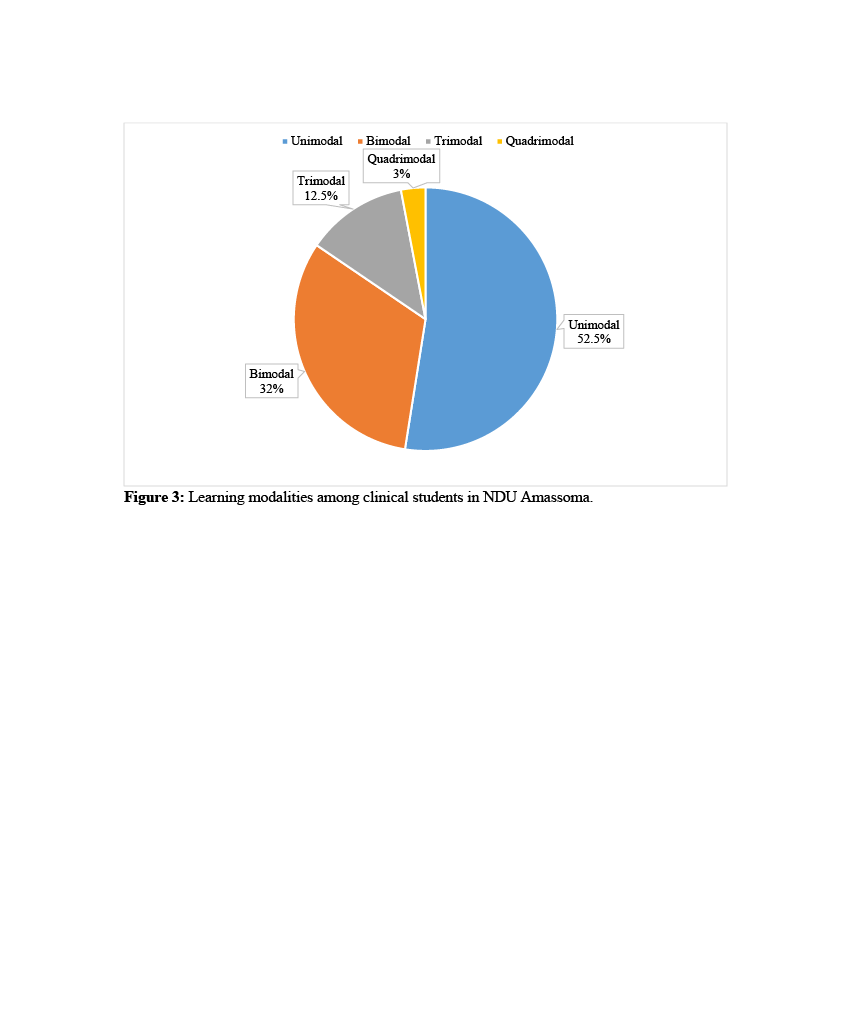

Supplement: Multimedia Appendix 1 [file formative-v10-e84089-s001.png]
