# Supplementary material for: Evidence on Learning Style Preferences Among Clinical Students in Nigeria Using the Visual, Aural, Read/Write, and Kinesthetic Model: Cross-Sectional Study
Source: JMIR Form Res. 2026 Jul 10;10:e84089. doi: 10.2196/84089 (PMC13359485; doi:10.2196/84089)
Supplement: Checklist 1 [file formative-v10-e84089-s002.doc]

STROBE Statement-Checklist of items that should be included in reports of ***cross-sectional studies***

|  | Item No | Recommendation |
| --- | --- | --- |
| **Title and abstract (Pp 1)** | 1 | (*a*) Indicate the study’s design with a commonly used term in the title or the abstract  Evidence on Learning Style Preferences Among Clinical Students in Nigeria Using the Visual, Aural, Read/Write, and Kinesthetic Model: Cross-Sectional Study |
|  |  | (*b*) Provide in the abstract an informative and balanced summary of what was done and what was found  The abstract provides a structured and balanced summary including background, objective, methods, results, and conclusions. |
| Introduction | | |
| Background/rationale (Pp 2) | 2 | Explain the scientific background and rationale for the investigation being reported  The introduction provides a clear scientific background, explaining the role of learning styles in medical education and the relevance of the VARK model. It highlights ongoing debates regarding the utility of learning styles and identifies a key evidence gap in sub-Saharan Africa, particularly among clinical students in Nigeria. The rationale for the study is justified by the need to generate context-specific evidence to inform curriculum design and improve clinical training in resource-constrained settings. |
| Objectives (Pp 2) | 3 | State-specific objectives, including any prespecified hypotheses  The study clearly states its objectives: to determine the predominant learning style preferences among clinical students using the VARK model and to examine associations with demographic and academic factors (sex, age, and year of study). Prespecified hypotheses are also provided, including a null hypothesis that learning preferences do not differ by these factors and an alternative hypothesis that they do. |
| Methods | | |
| Study design (Pp 3) | 4 | Present key elements of study design early in the paper  The key elements of the study design are presented early in the Methods section. The study is described as a cross-sectional, descriptive observational study designed to assess the distribution of learning preferences and their associations with selected demographic and academic variables. |
| Setting (Pp 3) | 5 | Describe the setting, locations, and relevant dates, including periods of recruitment, exposure, follow-up, and data collection  The study setting is clearly described as the College of Health Sciences, Niger Delta University (NDU), Amassoma, Bayelsa State, Nigeria. Relevant contextual details of the institution and learning environment are provided. The study period is specified as October to December 2021, and participants were recruited during scheduled academic activities. As a cross-sectional study, no follow-up period was applicable. |
| Participants (Pp 3) | 6 | (*a*) Give the eligibility criteria, and the sources and methods of selection of participants  The eligibility criteria are clearly defined. The study included clinical medical students (Years 4-6) who had completed preclinical training and were actively participating in clinical rotations, while those not available during the data collection period or not meeting these criteria were excluded. The source population comprised all clinical students at Niger Delta University (NDU), with a total population of 305 students. A proportionate sampling approach was used to allocate participants by year of study, followed by systematic random sampling using class registers as the sampling frame. Replacement was done by selecting the next available eligible student when necessary. |
| Variables (Pp 4) | 7 | Clearly define all outcomes, exposures, predictors, potential confounders, and effect modifiers. Give diagnostic criteria, if applicable  The outcome variables were learning style preferences measured using the VARK questionnaire (version 7.8), categorized into visual, auditory, read/write, and kinesthetic modalities, and further classified as unimodal or multimodal. Predictor variables included demographic and academic characteristics such as sex, age group, and year of study. These variables were also examined as potential effect modifiers. Learning style classification followed the standard VARK scoring protocol. No additional diagnostic criteria were applicable. |
| Data sources/ measurement (Pp 4) | 8* | For each variable of interest, give sources of data and details of methods of assessment (measurement). Describe comparability of assessment methods if there is more than one group  Data were collected using a structured, self-administered questionnaire comprising sociodemographic items and the standardized VARK questionnaire (version 7.8). Learning preferences were assessed using the validated 16-item VARK instrument, with scoring based on the official protocol. Sociodemographic variables were self-reported. The instrument was pretested, and reliability was confirmed with a Cronbach’s alpha of 0.78, alongside expert validation for content validity. The same measurement tools and procedures were applied uniformly across all participants, ensuring comparability. |
| Bias (Pp 4) | 9 | Describe any efforts to address potential sources of bias  To minimize potential sources of bias, several measures were implemented in the study design and data collection process. First, a cross-sectional survey design was used with inclusion of clinical students across multiple academic levels (400-600 level) within the same institution, reducing variability in exposure to different curricula during data collection. Second, a validated instrument (the VARK version 7.8 questionnaire) was used to ensure measurement validity and reduce information/measurement bias in assessing learning style preferences.  To further limit interviewer and observer bias, data were collected using a structured questionnaire, allowing participants to self-report their learning preferences independently. Standardized administration of the instrument ensured consistency across respondents.  In terms of analytical bias control, appropriate statistical tests (Chi-square and Fisher’s exact tests) were applied according to expected cell counts, reducing the risk of incorrect inference due to sparse data. Although multivariable modelling was initially considered, it was not performed due to categorical outcome structure and sample size limitations, thereby avoiding unstable estimates. Finally, the study acknowledges inherent limitations such as being conducted in a single institution, which may introduce selection bias and limit generalizability. |
| Study size (Pp 4) | 10 | Explain how the study size was arrived at  The study did not report a formal a priori sample size calculation. Instead, the sample size was determined based on the total accessible population of eligible clinical students (400-600 level) at the Niger Delta University during the study period (October to December 2021). All eligible students within this target population were approached for participation, and a total of 200 students who consented and completed the questionnaire constituted the final sample. Thus, the study size was primarily driven by feasibility and coverage of the available population at the time of data collection, rather than statistical power estimation or predetermined effect size assumptions. |
| Quantitative variables (Pp 4) | 11 | Explain how quantitative variables were handled in the analyses. If applicable, describe which groupings were chosen and why  In the study, quantitative variables were handled using both continuous and categorical approaches, depending on their analytical relevance. Age was treated as a continuous variable and summarized using measures of central tendency and dispersion (mean and standard deviation), specifically reported as mean age 25.1 years (SD = 3.9)  For inferential analysis, however, age was converted into categorical groupings (i.e., age groups). This categorization enabled the use of non-parametric statistical tests such as Pearson’s chi-square and Fisher’s exact tests to examine associations between demographic characteristics and learning style preferences. The decision to group age was methodologically appropriate because the primary outcome variable (learning style preference) was categorical, and chi-square analysis requires categorical inputs.  Other variables, such as sex and year of study, were inherently categorical and analyzed as such. Learning style preferences derived from the VARK instrument were also treated as categorical variables (e.g., visual, auditory, read/write, kinesthetic; unimodal vs. multimodal).  The grouping of quantitative variables (notably age) was therefore driven by:   - The need to ensure compatibility with categorical statistical tests (chi-square/Fisher’s exact), - The structure of the outcome variable (categorical learning preferences), - Sample size considerations and sparse data in some subcategories, which limited more complex modelling approaches such as multinomial regression.   Overall, the analytical handling reflects a pragmatic approach aligned with the study design, data structure, and statistical constraints. |
| Statistical methods (Pp 4) | 12 | (*a*) Describe all statistical methods, including those used to control for confounding  The statistical methods applied in the study were primarily descriptive and bivariate inferential techniques, selected to align with the categorical structure of the outcome variables and the study’s cross-sectional design.  Descriptive statistics were first used to summarize participant characteristics and learning style distributions. Continuous variables, such as age, were summarized using the mean and standard deviation, while categorical variables (e.g., sex, year of study, and learning style preferences) were presented as frequencies and percentages  For inferential analysis, Pearson’s chi-square test was employed to assess associations between categorical independent variables (sex, age group, and year of study) and learning style preferences. In instances where expected cell counts were small, Fisher’s exact test was used as an alternative to ensure statistical validity. These tests enabled the identification of statistically significant relationships between demographic factors and specific VARK modalities  Regarding control for confounding, the study initially specified a multivariable modelling strategy. However, this was not implemented due to key methodological constraints, including:   - The categorical nature of the primary outcome (learning style preferences), - Sparse data in several modality subcategories, - Insufficient sample size to support robust multinomial regression modelling.   As a result, no formal multivariable adjustment for confounding variables was conducted. Instead, potential confounding was indirectly explored through stratified bivariate analyses across key demographic variables (sex, age group, and year of study). While this approach allows for preliminary identification of associations, it does not provide the same level of control for confounding as multivariable regression techniques. Overall, the statistical approach reflects a constrained but methodologically coherent strategy given the data structure and sample limitations, though it limits causal inference and independent effect estimation. |
|  |  | (*b*) Describe any methods used to examine subgroups and interactions  Subgroup analyses in the study were conducted using stratified bivariate methods to explore how learning style preferences varied across key participant characteristics. Specifically, the sample was disaggregated by sex, age group, and year of study, and associations with VARK learning style categories (visual, auditory, read/write, kinesthetic; unimodal vs. multimodal) were examined using Pearson’s chi-square and Fisher’s exact tests. This approach enabled the identification of differences in learning preferences within defined subpopulations, for example, variations in visual preference by sex and read/write preference by year of study. However, no formal statistical interaction analyses were performed. That is, the study did not include interaction terms within a multivariable modelling framework (e.g., sex × year of study) to assess effect modification. This limitation is attributable to the absence of multivariable regression analysis, which was not implemented due to the categorical structure of the outcome variables, sparse data in several categories, and sample size constraints. Consequently, subgroup differences were assessed independently rather than jointly, and potential interaction effects between variables could not be formally tested. While the stratified analyses provide useful descriptive and inferential insights, they do not allow for robust evaluation of effect modification or combined influences of multiple factors. |
|  |  | (*c*) Explain how missing data were addressed  The study did not explicitly report the presence of missing data or describe any formal methods for handling missing values. All analyses appear to have been conducted on a complete-case basis, as evidenced by the consistent use of a total sample size of 200 participants across descriptive and inferential results.  This suggests that either:   - There were no missing data for the variables included in the analysis, or - Any incomplete responses were excluded prior to analysis (i.e., listwise deletion), although this was not explicitly stated.   No imputation techniques (such as mean substitution, multiple imputation, or model-based approaches) were applied, and no sensitivity analyses were conducted to assess the potential impact of missing data on the study findings. Overall, the handling of missing data is implicit rather than formally addressed, which represents a reporting limitation under STROBE guidelines, as it restricts the ability to assess potential bias arising from incomplete observations. |
|  |  | (*d*) If applicable, describe analytical methods taking account of the sampling strategy  The study did not report the use of any complex sampling strategy that would require specialized analytical adjustments. Participants were recruited as a defined group of clinical students (400-600 level) from a single institution using a cross-sectional survey approach, and the analysis treated the sample as a simple, unweighted cohort.  Accordingly, no analytical techniques were applied to account for sampling design features such as stratification, clustering, or unequal probabilities of selection. There was also no use of survey weights, design effects, or multilevel modelling approaches. All statistical analyses (descriptive statistics and chi-square/Fisher’s exact tests) assumed independence of observations and equal contribution of each participant to the estimates.  This approach is methodologically consistent with a convenience or census-like sample within a single academic setting. However, it implies that the findings are not adjusted for potential sampling biases and may have limited generalizability beyond the study population. |
|  |  | (*e*) Describe any sensitivity analyses  No sensitivity analyses were reported or conducted in the study. The analytical approach was limited to descriptive statistics and bivariate tests (Pearson’s chi-square and Fisher’s exact), without additional procedures to test the robustness of the findings under alternative assumptions or analytical specifications.  In particular, the study did not perform sensitivity checks such as:   - Re-analysis using different categorizations of variables (e.g., alternative age groupings), - Assessment of the impact of excluding sparse categories, - Comparison of results under different statistical test assumptions, - Evaluation of potential bias due to unmeasured confounding or missing data.   The absence of sensitivity analyses is consistent with the study’s methodological constraints, including the relatively small sample size, categorical structure of the outcome variables, and lack of multivariable modelling. However, it represents a limitation, as the robustness and stability of the reported associations cannot be formally assessed. |
| Results (Pp 4) | | |
| Participants (Pp 4-10) | 13* | (a) Report numbers of individuals at each stage of study—eg numbers potentially eligible, examined for eligibility, confirmed eligible, included in the study, completing follow-up, and analysed  The study provides limited but partially sufficient information regarding the number of participants at different stages of the research process.  A total of 200 clinical students (400-600 level) were included in the study and formed the final analytical sample. All 200 participants appear to have completed the survey and were included in both descriptive and inferential analyses, as the reported results consistently reference this total sample size.  However, the study does not report:   - The number of individuals initially approached or potentially eligible for participation, - The number assessed for eligibility, - The number excluded (with or without reasons), - Any losses prior to analysis.   Given the cross-sectional design and questionnaire-based data collection, there was no follow-up phase; therefore, stages such as “completing follow-up” are not applicable.  Overall, while the final sample size and number analysed are clearly stated (n = 200), the earlier stages of participant flow are not described. This represents a limitation in reporting transparency according to STROBE guidelines, as it restricts assessment of potential selection bias and study representativeness. |
|  |  | (b) Give reasons for non-participation at each stage  The study does not report any information on non-participation or provide reasons for non-participation at any stage of the research process. Specifically, there is no documentation of:   - The number of eligible students who declined participation, - Reasons for refusal or non-response, - Any exclusions due to incomplete or invalid questionnaire responses.   Given that the final sample of 200 participants is consistently used in all analyses, it is possible that only fully completed questionnaires were included (implying implicit exclusion of incomplete responses), but this is not explicitly stated.  The absence of reported reasons for non-participation limits the ability to assess potential non-response bias or systematic differences between participants and non-participants. This constitutes a reporting gap under STROBE guidelines and affects the transparency and interpretability of the study’s sampling process. |
|  |  | (c) Consider use of a flow diagram  Given the cross-sectional design and the reporting of a single final sample size (n = 200), the inclusion of a flow diagram would have improved transparency by clearly documenting:   - The initial pool of potentially eligible clinical students, - The number approached and assessed for eligibility, - Any exclusions (with reasons), - The final number included in the analysis.   Although a flow diagram is not strictly mandatory for cross-sectional studies, its inclusion is recommended under STROBE guidelines to enhance clarity and allow readers to better assess potential selection bias and the completeness of data collection.  In this case, the absence of a flow diagram reflects limited reporting of participant flow and recruitment processes. |
| Descriptive data (Pp 4-10) | 14* | (a) Give characteristics of study participants (eg demographic, clinical, social) and information on exposures and potential confounders  The study provides key demographic and academic characteristics of the participants, along with information on the primary exposure variables and potential confounders relevant to the analysis.  A total of 200 clinical students participated in the study. The mean age was 25.1 years (SD = 3.9), indicating a relatively young adult population typical of clinical training cohorts. Participants were further categorized into age groups for inferential analysis. In terms of sex distribution, 107 participants (53.5%) were male, while the remainder were female. Academic level was also reported, with participants drawn from 400–600 levels, representing different stages of clinical training.  The primary outcome (often treated analytically as the dependent variable) was learning style preference, measured using the VARK model. This included four modality categories, visual, auditory, read/write, and kinesthetic, as well as classification into unimodal (52.5%) and multimodal (47.5%) preferences. Kinesthetic (60.5%) and auditory (55.0%) preferences were the most prevalent, followed by read/write (34.0%) and visual (18.0%).  Key exposure variables included demographic and academic factors such as sex, age group, and year of study. These variables were selected due to their potential influence on learning preferences and were examined in bivariate analyses.  Potential confounders were limited to these same measured variables (sex, age group, and year of study), as no additional social, socioeconomic, or clinical variables (e.g., prior educational background, academic performance, or learning environment factors) were collected or reported. Furthermore, no multivariable analysis was conducted to formally adjust for confounding. Overall, while the study adequately reports basic demographic and academic characteristics, the range of potential confounders is relatively narrow, which may limit the depth of interpretation regarding factors influencing learning style preferences. |
|  |  | (b) Indicate number of participants with missing data for each variable of interest  The provided study does not report any missing data for any of the variables of **interest** (e.g., sex, age, year of study, learning style preferences). All 200 participants were included in the analysis, and the results are presented with complete denominators (e.g., 200 for all reported proportions).  Therefore, based on the study, it can be stated that:   - No missing data were explicitly indicated for any study variable - Analyses appear to have been conducted on a complete-case basis (N = 200 for all variables reported) |
| Outcome data (Pp 4-10) | 15* | Report numbers of outcome events or summary measures  The study included 200 clinical students, with all participants completing the VARK questionnaire, yielding complete outcome data for learning style preference categories. No missing outcome data were reported for the primary variables of interest. The outcome distribution showed that 105/200 (52.5%) of participants preferred unimodal learning styles, while 95/200 (47.5%) demonstrated multimodal preferences. With respect to specific VARK modalities, kinesthetic learning was the most frequently dominant preference (121/200; 60.5%), followed by auditory (110/200; 55.0%), read/write (68/200; 34.0%), and visual (36/200; 18.0%). These values represent unadjusted descriptive summary measures of the primary outcome. |
| Main results (Pp 4-10) | 16 | (*a*) Give unadjusted estimates and, if applicable, confounder-adjusted estimates and their precision (eg, 95% confidence interval). Make clear which confounders were adjusted for and why they were included. All reported estimates are unadjusted proportions and chi-square-based associations. No multivariable regression analyses were conducted; therefore, no confounder-adjusted estimates or confidence intervals are available.  Bivariate analyses showed:   - Visual learning preference was significantly higher among male students (χ² = 4.49, p = 0.034). - Read/write preference varied significantly by year of study (χ² = 8.29, p = 0.016). - No statistically significant association was observed between age group and learning style preference.   No confounders (e.g., age, sex, year of study) were adjusted for in multivariable models. This was explicitly due to the categorical structure of the outcomes, sparse cell counts in some VARK categories, and limitations in sample size that precluded reliable multinomial modelling. |
|  |  | (*b*) Report category boundaries when continuous variables were categorized  Age was originally measured as a continuous variable (mean 25.1 years, SD 3.9). However, the study reports that age was also analyzed in grouped categories for inferential testing (exact cut-off boundaries for these age groups were not specified in the available dataset extract). Other variables, including sex (male/female), year of study (400-600 level), and learning style preference (visual, auditory, read/write, kinesthetic; unimodal vs multimodal), were inherently categorical and required no further categorization boundaries. |
|  |  | (*c*) If relevant, consider translating estimates of relative risk into absolute risk for a meaningful time period  This was a cross-sectional study; therefore, relative risk estimates were not calculated. The findings are already presented as absolute proportions (e.g., prevalence of kinesthetic preference = 60.5%, auditory = 55.0%), which directly represent absolute outcome frequencies within the study population. |
| Other analyses (Pp 4-10) | 17 | Report other analyses done, e.g., analyses of subgroups and interactions, and sensitivity analyses  Subgroup analyses were conducted using chi-square tests:   - Sex-based subgroup analysis showed a significant association with visual learning preference. - Year of study subgroup analysis showed a significant association with read/write preference. - No interaction analyses between demographic variables (e.g., sex × year of study) were reported. - No sensitivity analyses were conducted or reported.   A prespecified multivariable modelling strategy was considered but not implemented due to sparse categorical data distribution and insufficient sample size for stable multinomial regression estimation. |
| Discussion | | |
| Key results (Pp 10-12) | 18 | Summarise key results with reference to study objectives  The primary objective was to determine the predominant learning style preferences among clinical students and examine demographic influences. The study found that kinesthetic and auditory learning styles were most prevalent, with nearly half of respondents exhibiting multimodal preferences. Significant associations were observed between sex and visual preference, as well as year of study and read/write preference. Age was not significantly associated with learning style distribution. Overall, the findings indicate a strong preference for experiential and auditory learning modalities among clinical students in the study setting. |
| Limitations (Pp 12) | 19 | Discuss limitations of the study, taking into account sources of potential bias or imprecision. Discuss both direction and magnitude of any potential bias  The study has several limitations. First, its cross-sectional design limits causal inference between demographic factors and learning style preferences. Second, reliance on self-reported VARK inventory responses introduces the potential for reporting and social desirability bias, which may overestimate alignment with perceived “ideal” learning styles. Third, the use of a single-institution sample restricts generalizability to other medical schools in Nigeria or sub-Saharan Africa. This introduces potential selection bias, with unknown direction but likely limited external validity. Fourth, the relatively small sample size (n = 200) and sparse distribution across some VARK categories reduced statistical power and prevented multivariable adjustment. This increases the risk of residual confounding in observed bivariate associations, potentially inflating or obscuring true relationships between demographic variables and learning preferences. Finally, the lack of multivariable modelling means that observed associations (e.g., sex and visual preference) may be partially confounded by unmeasured or uncontrolled variables such as prior educational exposure or learning environment, potentially biasing effect estimates in an unknown direction. |
| Interpretation (Pp 12) | 20 | Give a cautious overall interpretation of results considering objectives, limitations, multiplicity of analyses, results from similar studies, and other relevant evidence  The study set out to identify dominant learning style preferences among clinical students and explore demographic influences. The findings suggest a predominance of kinesthetic and auditory learning preferences, with nearly half of the participants indicating multimodal learning styles. Statistically significant associations were observed between sex and visual preference, and between year of study and read/write preference. However, these results should be interpreted cautiously. First, the study relied on self-reported VARK classifications, which are subject to measurement limitations and ongoing debate regarding the construct validity of learning-style frameworks. Second, the absence of multivariable adjustment limits the ability to infer independent associations, raising the possibility of residual confounding. Third, multiple bivariate tests were conducted (e.g., across sex, age, and year of study against multiple learning modalities), increasing the risk of type I error due to multiplicity of analyses. Some statistically significant findings may therefore reflect chance rather than true underlying relationships.  When considered alongside existing literature, which similarly reports mixed and context-dependent learning-style distributions among medical students, the results align broadly with studies suggesting a preference for active, experiential learning in clinical populations. However, other educational research questions the instructional utility of learning-style categorization, suggesting that observed preferences may not necessarily translate into improved learning outcomes when teaching is tailored accordingly. Overall, the findings provide useful descriptive insight into learner preferences but should not be over-interpreted as evidence for rigid pedagogical matching. |
| Generalisability (Pp 12) | 21 | Discuss the generalisability (external validity) of the study results  The external validity of the study is limited. The sample was drawn from a single medical school in Nigeria (Niger Delta University), and thus reflects a specific institutional, cultural, and curricular context. Learning preferences may differ across institutions due to variations in teaching methods, clinical exposure, educational resources, and student demographics.  Additionally, the sample size (n = 200) and the restriction to clinical-level students (400–600 level only) further constrain generalisability to preclinical students or those in other health disciplines. Therefore, while the findings may apply to similar Nigerian or sub-Saharan African medical training environments, caution is warranted in extrapolating to broader national or international populations. |
| Other information | | |
| Funding (Pp 15) | 22 | Give the source of funding and the role of the funders for the present study and, if applicable, for the original study on which the present article is based  The study explicitly reports that it received no specific grant from any funding agency in the public, commercial, or not-for-profit sectors. As such, there was no external funding source supporting the design, data collection, analysis, or interpretation of the study. Consequently, there was no role of funders in any aspect of the research process, including study conception, methodology, data analysis, manuscript preparation, or publication decisions. |

*Give information separately for exposed and unexposed groups.

**Note:** An Explanation and Elaboration article discusses each checklist item and gives methodological background and published examples of transparent reporting. The STROBE checklist is best used in conjunction with this article (freely available on the Web sites of PLoS Medicine at http://www.plosmedicine.org/, Annals of Internal Medicine at http://www.annals.org/, and Epidemiology at http://www.epidem.com/). Information on the STROBE Initiative is available at www.strobe-statement.org.
